# Entanglements via Slip Springs with Soft, Coarse-Grained Models for Systems Having Explicit Liquid–Vapor Interfaces

**DOI:** 10.1021/acs.macromol.3c00960

**Published:** 2023-09-01

**Authors:** Ludwig Schneider, Juan J. de Pablo

**Affiliations:** † Pritzker School of Molecular Engineering, University of Chicago, 5740 S. Ellis Avenue, Chicago, Illinois 60637-1403, United States; ‡ Argonne National Laboratory, 9700 S. Cass Avenue, Lemont, IL 60439, United States

## Abstract

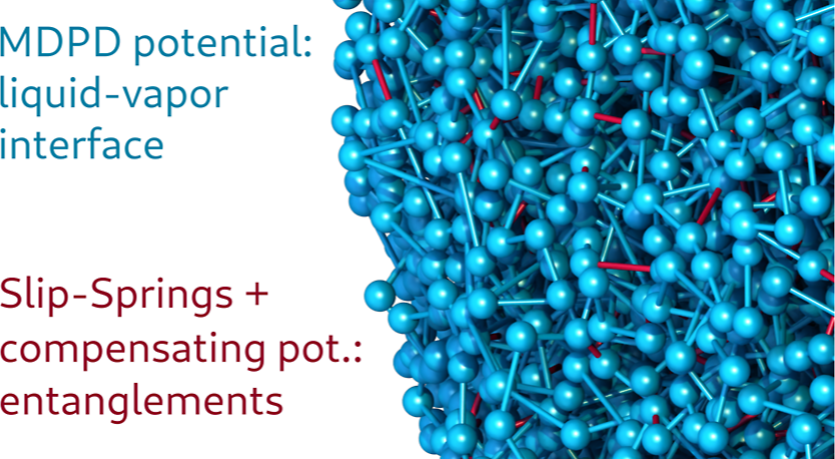

Recent advances in nano-rheology require that new techniques and models be developed to precisely describe the equilibrium and non-equilibrium characteristics of entangled polymeric materials and their interfaces at a molecular level. In this study, a slip-spring (SLSP) model is proposed to capture the dynamics of entangled polymers at interfaces, including those between liquids, liquids and vapors, and liquids and solids. The SLSP model employs a highly coarse-grained approach, which allows for comprehensive simulations of entire nano-rheological characterization systems using a particle-level description. The model relies on many-body dissipative particle dynamics (MDPD) non-bonded interactions, which permit explicit description of liquid–vapor interfaces; a compensating potential is introduced to ensure an unbiased representation of the shape of the liquid–vapor interface within the SLSP model. The usefulness of the proposed MDPD + SLSP approach is illustrated by simulating a capillary breakup rheometer (CaBR) experiment, in which a liquid droplet splits into two segments under the influence of capillary forces. We find that the predictions of the MDPD + SLSP model are consistent with experimental measurements and theoretical predictions. The proposed model is also verified by comparison to the results of explicit molecular dynamics simulations of an entangled polymer melt using a Kremer–Grest chain representation, both at equilibrium and far from equilibrium. Taken together, the model and methods presented in this study provide a reliable framework for molecular-level interpretation of high-polymer dynamics in the presence of interfaces.

## Introduction

Molecular entanglements in polymeric materials give rise to unique viscoelastic properties. Individual macromolecules can reach contour lengths of multiple microns, and the resulting topological constraints that arise in condensed polymeric phases lead to long-term relaxation processes that are very different from those observed in simpler, small-molecule liquids.^1^ These properties are well known for bulk materials; however, less is known about the effects of interfaces on entangled polymer dynamics. The advent of nano-rheology^2,3^ presents intriguing opportunities to examine the role of entanglements in situations where confinement restricts the material to length scales comparable to the size of individual molecules. Understanding that role could, in fact, help nano-rheology measurements become a standard tool for the characterization of bulk rheology from ultra-small samples. As discussed in this work, coarse-grained simulations provide a unique means to represent entire nano-rheology systems, such as droplets or filaments, while retaining molecular information explicitly. Slip-spring (SLSP) models^4−9,9−14,14−20^ have gradually been developed to describe the dynamics of high-molecular-weight entangled polymeric liquids. A particle-level description ensures that the dynamics of the system are captured at a molecular level, and SLSPs are introduced to mimic the effects of entanglements within the context of a highly coarse-grained representation.^15,17,21−28^ More specifically, a high degree of coarse-graining is generally accompanied by relying on soft non-bonded interaction potentials that do not prevent chain crossing; the artificial springs encoded in the SLSP representation serve to reintroduce topological constraints.

Much of the literature on SLSP models has focused on establishing their correct asymptotic behavior (*e.g.*, by showing that the power–law dynamics predicted by tube models can be reproduced^4,5,8,29^). More recent efforts have sought to introduce systematic procedures to parameterize models for specific chemical systems or more complex molecular architectures.^15,17,30,31^ Importantly, the existing body of work on SLSP models has been limited to studies of the bulk properties of polymers or polymer nano-composites.^32−34^

We adopt an established many-body dissipative particle dynamics (MDPD) approach^35−38^ to study entanglements within a coarse-grained representation that permits the explicit formation of liquid–vapor interfaces.^35−38^ This technique features a local density-dependent repulsion term that transforms simple pairwise interactions into a many-body potential. This empirical concept gives rise to an equation of state that includes liquid–vapor coexistence and that features a sharp interface between the two phases, which is characteristic of the length scale of coarse-grained models. This approach leads to relatively efficient simulations because a liquid–vapor interface can form without the need for computationally expensive long-range attractions. This concept has been steadily refined over the past two decades.^39,40^ A related application of this approach can be found in the works of Müller *et al.*, who applied the MDPD in examples where the vapor phase represents an implicit solvent for membranes and polymer brushes, respectively.^41,42^

In this study, we combine a SLSP model with an established MDPD model to arrive at a new approach capable of describing entanglement effects in systems with interfaces at a coarse-grained level. The integration of SLSPs and an explicit liquid–vapor interface represents a development that provides new opportunities for comprehensive investigations of polymer nano-rheology such as CaBR *in silico*. We would like to note that our CaBR simulations deviate from the conventional capillary breakup extensional rheometer (CaBER) experiments in one important aspect: we do not increase the distance between the two suspending planes. Our expectation is that this modification will help avoid some of the artifacts that arise from artificial chain extensions, which are often encountered in simulations. Detailed discussions and explanations regarding this approach are provided in the subsequent sections. This development obviates the need for dummy particles, for example, which were used before to represent the gas phase as a structureless liquid,^43,44^ or the need for explicit confinement.^45^

## Methods

Several SLSP implementations for shear flow simulations have relied on a dissipative particle dynamics (DPD) thermostat^46,47^ to control temperature. Such a thermostat conserves particle momentum locally, thereby providing a correct description of hydrodynamic effects.

In the DPD formalism, non-bonded interactions are represented by a simple quadratic repulsion force of the form
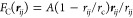
1

Here, the positive parameter *A* regulates the compressibility, ***r***_*ij*_ is the vector pointing from particle *i* to particle *j*, and *r*_*ij*_ is the length of this vector. Long-range attractive interactions are not included.

In contrast, in the MDPD potential,^35,36,39^ non-bonded interactions are divided into two parts: eq 1 acts as an attractive potential when the *A* parameter has a negative value, and a second many-body force contribution is added, which depends on the instantaneous density  around particles *i*
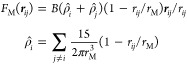
2

Here, *B* regulates the repulsion strength, and ρ_*i*_ describes the total local density surrounding particle *i*. Note that the cutoff distance is different for the two potential parts, such that *r*_M_ ≠ *r*_c_.

In this work, we use the values *A* = 40 and *B* = 10, and cutoff distances *r*_c_ = σ > *r*_M_ = 0.75σ, which correspond to a state point along the liquid–vapor coexistence curve, with a liquid branch density of ρ = (7.1 ± 0.2)σ^–3^, where σ is the internal length unit of the DPD model.

**Figure 1:**
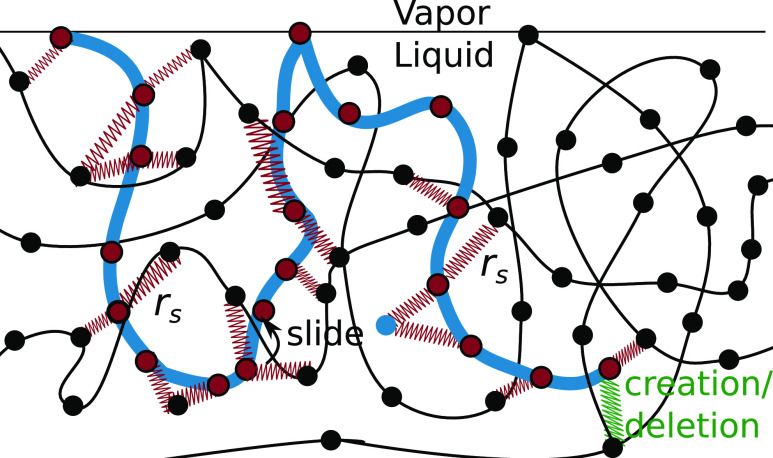
Schematic representation of the SLSP model in the vicinity of a liquid–vapor interface, including MDPD non-bonded interactions. The model incorporates topological constraints through SLSPs that connect the blue chain to the neighboring black chains. The vapor phase does not impose any additional topological constraints and reflects the chain contour.

The remaining aspects of the model are adopted from refs (4)(15), and (19). A schematic representation of the SLSP model in the presence of a liquid–vapor interface is shown in Figure 1.

Polymer chains in this study are modeled as beads connected by harmonic bonds, described by the potential  with a spring constant of *k* = 16*k*_B_*T*/(3σ^2^). The average bond extension is . Entanglements are introduced *via* finite extensible nonlinear elastic (FENE) SLSPs using a grand canonical ensemble, where the potential is given by , with *k*_ss_ = *k* and *r*_ss_ = σ. The average number of SLSPs is controlled by a chemical potential μ and a corresponding fugacity ; the average number of SLSPs is related to *z* by ⟨*n*_ss_⟩ ∝ *z*.^4^

The equations of motion are integrated using a time step of Δ*t* = 10^–3^τ, with τ being the internal time unit of the DPD model. Our simulations have been implemented in HOOMD-blue version 2.9.7 with custom plug-ins.^48−51^ In order to capture the dynamic nature of entanglements, we refresh the SLSP configuration using a slide move every 10^2^ time steps. Furthermore, we perform creation and deletion moves at the chain ends every 10^3^ time steps to simulate the process of tube renewal.

Our chosen update frequency for the SLSP represents a strategic balance between computational efficiency and the necessity to preserve the equilibrium between the SLSP connections and the polymer chains. This frequency ensures that the SLSP configurations are refreshed before the average particle displacement exceeds the extension of the SLSP.

Note that this choice does exert an influence on the dynamics of the model, but its impact can be successfully counterbalanced, especially over extended timescales. Specifically, this counterbalance can be achieved by fostering synergistic coordination between the Kremer–Grest (KG) and SLSP models. A demonstration of this compensatory mechanism in action can be found in prior literature, as referenced in ref (19).

Additional effective attractions induced by SLSPs are compensated by a potential of the form *V*_comp_(***r***) = *k*_B_*Tz* exp[−β*V*_ss_(***r***)].^4^

**Figure 2:**
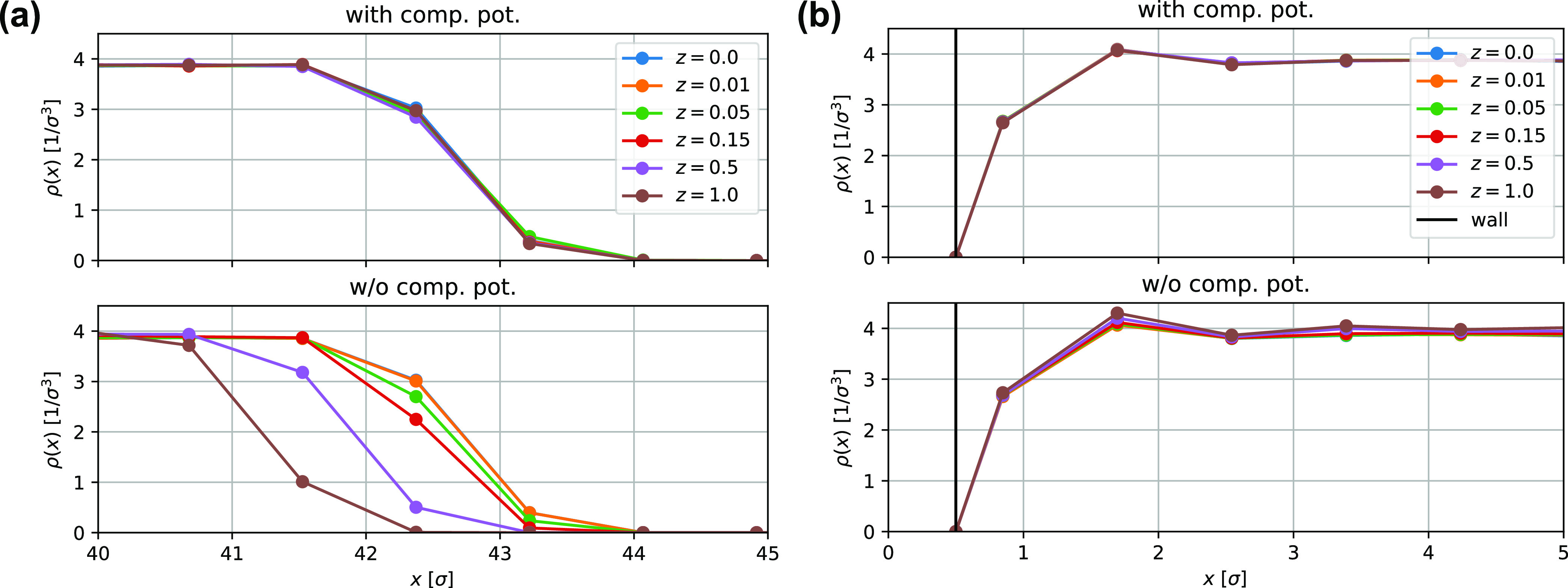
Density profile of a liquid–vapor system with and without the compensating potential, depicted in the top and bottom panels, respectively. Different colors are used to represent the density of differently entangled materials, achieved by varying the fugacity *z*. The figure illustrates that the compensating potential is a crucial feature of this model as it avoids undesired artifacts near the interfaces. (a) Density profile of a liquid–vapor interface under varying numbers of entanglements: without the compensating potential, both the position and shape of the interface are affected, leading to undesired changes in the system. (b) Wall–liquid interface for varying degrees of entanglement: without the compensating potential, the arrangement of particles near the wall is altered.

The compensating potential described in ref (4) plays a crucial role in liquid–vapor interfaces. While a simple pressure correction has been used in past studies to mitigate the attractive interaction of SLSPs in bulk systems,^8,12,14^ it cannot be applied directly to liquid–vapor systems without affecting the liquid–vapor coexistence. To avoid such issues, we adopt an explicit compensating potential, as detailed in ref (4), which ensures that the density of SLSP does not impact the liquid–vapor or liquid–wall interface position or shape, as illustrated in Figure 2. This separation of dynamic and static properties enables independent tuning of these properties during the top-down coarse-graining process.

To model a substrate in our system, we introduce a Lennard-Jones (LJ)-type wall with the potential form of , where *r*_w_ describes the distance between the particles and the wall, with parameters ε_wD,LJ_ = 10*k*_B_*T* and σ_wD,LJ_ = 3/4σ and a cutoff distance of *r*_wD,LJ_ = σ. Note that we rely on the MDPD model, which is different from the more common DPD model. The MDPD model creates a realistic packing close to the interface and compensates for the “missing neighbor” effect. It should be noted, however, that this compensation is not explicitly necessary, as discussed in ref (52).

As shown in Figure 2, the high degree of coarse-graining in the MDPD model requires a flat profile. Atomistic packing only appears at smaller length scales, which explains the absence of density peaks close to the interface.

### Kremer–Grest Model

To examine the validity of the SLSP model, in what follows, we compare its results to those of an entangled KG model.^53^ The LJ non-bonded interactions’ hard repulsion, combined with a restricted FENE potential for the backbone interactions, prevents chain backbones from crossing each other, giving rise to direct entanglements. For this purpose, we use a chain length of *N*_LJ_ = 128 beads.

We make two adjustments to the original KG model to improve our simulation. First, we introduce an additional angle potential between all consecutive bonds along the chain backbones of the form . This slightly stiffens the polymer chain and promotes entanglements.^54^ To achieve this, we set the parameters *k*_θ_ = 0.2*k*_B_*T* and θ_0_ = π. Second, we do not cut off the LJ potential early for pure repulsion; instead, we select a cutoff length of *r*_LJ,cut_ = 1.5σ_LJ_ and an energy well depth of 2.25ε_LJ_. The combination adds a longer-range attraction to the system, allowing us to simulate an explicit liquid–vapor interface, as in the MDPD model.

To obtain an equilibrium density close to the density reported in the original KG model, we set the pressure to 0 and obtained a density of , where σ_LJ_ is the internal length scale of the LJ model. To integrate these KG models, we used a time step of Δ*t* = 10^–4^τ_LJ_, where τ_LJ_ is the internal timescale of the LJ model, and equilibrated them *via* a soft push-off method similar to the one reported in ref (54).

We characterized the entanglements in this KG model using the established Z1+ algorithm.^55^ The algorithm identified the kinks in the chains’ primitive path, and the results are presented in Table 1. The results demonstrate that the system is entangled as anticipated and is consistent with similar models reported in the literature.^54^

**Table 1:** Entanglement Characteristics of the KG Chain Model with Implicit Entanglements Obtained through the Application of the Z1+ Algorithm,^55^^a^

average kink number per chain ⟨*Z*⟩	4.9
*N*_e_ kinks (classical)	21.5
*N*_e_ kinks (modified)	25.9
*N*_e_ coil (classical)	42.1
*N*_e_ coil (modified)	57.1

a The algorithm identifies kinks and coils in both the classical and modified methods, following the method described in ref (56) to determine the distance between entanglements, represented by *N*_e_.

This model provides implicit entanglements and allows for simulating an explicit liquid–vapor interface, making it an ideal candidate for comparing the proposed MDPD and SLSP models. To create a corresponding system in the MDPD model, we match the invariant degree of polymerization between the two systems. For example, the KG model has an invariant degree of polymerization , where *R*_e0_ is the theoretically expected root-mean-squared end-to-end distance of the polymer in the bulk. We can approximate this value in the MDPD model by choosing a discretization of *N*_SLSP_ = 12, which means that approximately 10 KG segments are modeled with a single MDPD bead. Although this discretization is relatively small, it is sufficient to achieve Gaussian chain statistics and necessary to limit the computational demands of the KG chain model.

To facilitate a consistent comparison between the SLSP and MDPD models, it is important to align their respective timescales and fugacities. We do so by relying on a published method that enables linking higher-resolution models to SLSP models.^15^ More specifically, we match the end-to-end vector correlation of the two models in the bulk and establish the optimal fugacity that aligns the correlations using this technique.

We carefully adjust the curves of the two models, considering both long and short timescales. Using these two points of reference, we can determine the optimal time shift factor τ and the optimal fugacity, which is found to be *z* = 0.225 for our system. This allows us to establish a correspondence of the decay of the end-to-end autocorrelation function of the two models over more than 2 orders of magnitude. This calibration ensures that both models operate on the same timescale, thereby permitting direct comparisons. For details, readers are referred to the Supporting Information and ref (15).

## Results and Discussion

Having established the connections between the KG and SLSP + MDPD models, we now compare the results of the two models and assess the validity of the SLSP + MDPD model in systems that exhibit a liquid–vapor interface.

### Planar Liquid–Vapor Interface

The first step is to investigate the similarity between the interaction of the SLSP models and implicit entanglements with the liquid–vapor interface. We identify the entanglement locations relative to the interface in a freestanding liquid–vapor interface using the Z1+ algorithm.^55^ Although there is no direct correspondence between SLSPs and kinks in the primitive path, as the SLSPs are not necessarily seen as pairwise entanglements and their dynamical effects depend on the model parameters,^19^ comparing the positions of the kinks and SLSPs offers a qualitative validation of the SLSP model.

**Figure 3:**
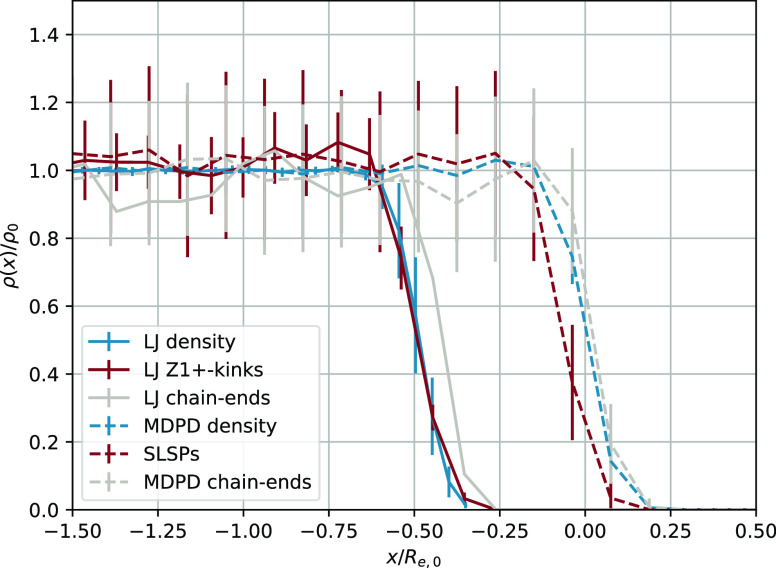
Comparison of the concentration profiles of SLSPs and kinks in the primitive path of the KG chains in the proximity of a liquid–vapor interface. The profiles were determined *via* the Z1+ algorithm^55^ and were shifted along the abscissa for clarity. To facilitate the comparison, the profiles were normalized to overlap with the density and chain end density profiles. Both models exhibit similar qualitative features, including a slight drop in the density of both kinks and SLSPs close to the interface. This drop is a consequence of the fact that chains near the interface can only entangle with chains in the bulk. This agreement suggests that the static properties of entanglements are preserved in the MDPD plus SLSP model. Also, note the enrichment of the chain ends close to the interface observed in both models.

In Figure 3, we compare the concentration profiles of SLSPs and kinks in the primitive path of the KG chains. As expected, the profile is flat in the bulk region. Near the interface, we observe a slight decrease in the density of kinks and SLSPs. This decrease results from the fact that a chain near the interface can only entangle with chains in the bulk region (since there are no chains on the other side).

To capture the dynamics of entanglements, it is critical that the concentration of polymer chain ends be taken into account, as entanglements occur exclusively at the ends of chains. Our analysis reveals a slight enrichment of chain ends in the vicinity of the liquid–vapor interface, which is evident from their slower decay compared to the overall density. This enrichment of chain ends near the interface has been previously reported in the literature^57−59^ and is consistent with the results from both the KG model and the MDPD plus SLSP model.

Our simulations indicate that the concentration profile of SLSPs closely reproduces that of kinks in a highly resolved model. This finding serves to validate the use of the MDPD plus SLSP model for studies of entanglement network structure and dynamics.

Our results for a liquid–vapor interface are similar to those reported by Kirk *et al.*(60−63) for polymers confined by a wall. Those studies demonstrated that the interface has no significant impact on the number of kinks near the interface and that a SLSP model can reproduce these findings accurately.

The impact of entanglements on dynamic properties is of considerably more consequence than a static comparison of the empirical position of entanglements between the two models. For that reason, in the next section, we consider an inherently dynamic system that features a prominent liquid–vapor interface.

### Simulated Capillary Breakup Rheometer

Entanglements are dynamic entities, and, as pointed out before, one-to-one comparisons to kinks in the primitive path can be challenging.^19,57^ To test the dynamics of the proposed model against those of the KG model, we focus on a rheology measurement technique known as a capillary breakup rheometer (CaBR).^64,65^ Earlier works^15^ have already established that the SLSP model can accurately reproduce entanglement dynamics in the bulk. The current CaBR setup serves to demonstrate that this is also the case in the presence of a non-negligible liquid–vapor interface.

In this technique, a polymer droplet is confined between two wetting surfaces. Capillary forces then act on the droplet, elongating it until the droplet breaks into two halves. In experiments, during the breakup, the minimum extension of the neck *R*(*t*) that separates the droplet’s halves is tracked with a camera. The balance of capillary forces exerted by the interface as well as the flow inside the droplet determine how the neck shape evolves (the neck size) over time.^65^ Initially, the apparent viscosity η balances the surface tension Γ, resulting in a linear decay of *R*(*t*) ∝ −*t*Γ/(6η). In the terminal stage, the disentanglement process dominates the dynamics, and the neck decays exponentially with the disentanglement time, . This behavior has been documented in experiments with polymer solutions.^66^

**Figure 4:**
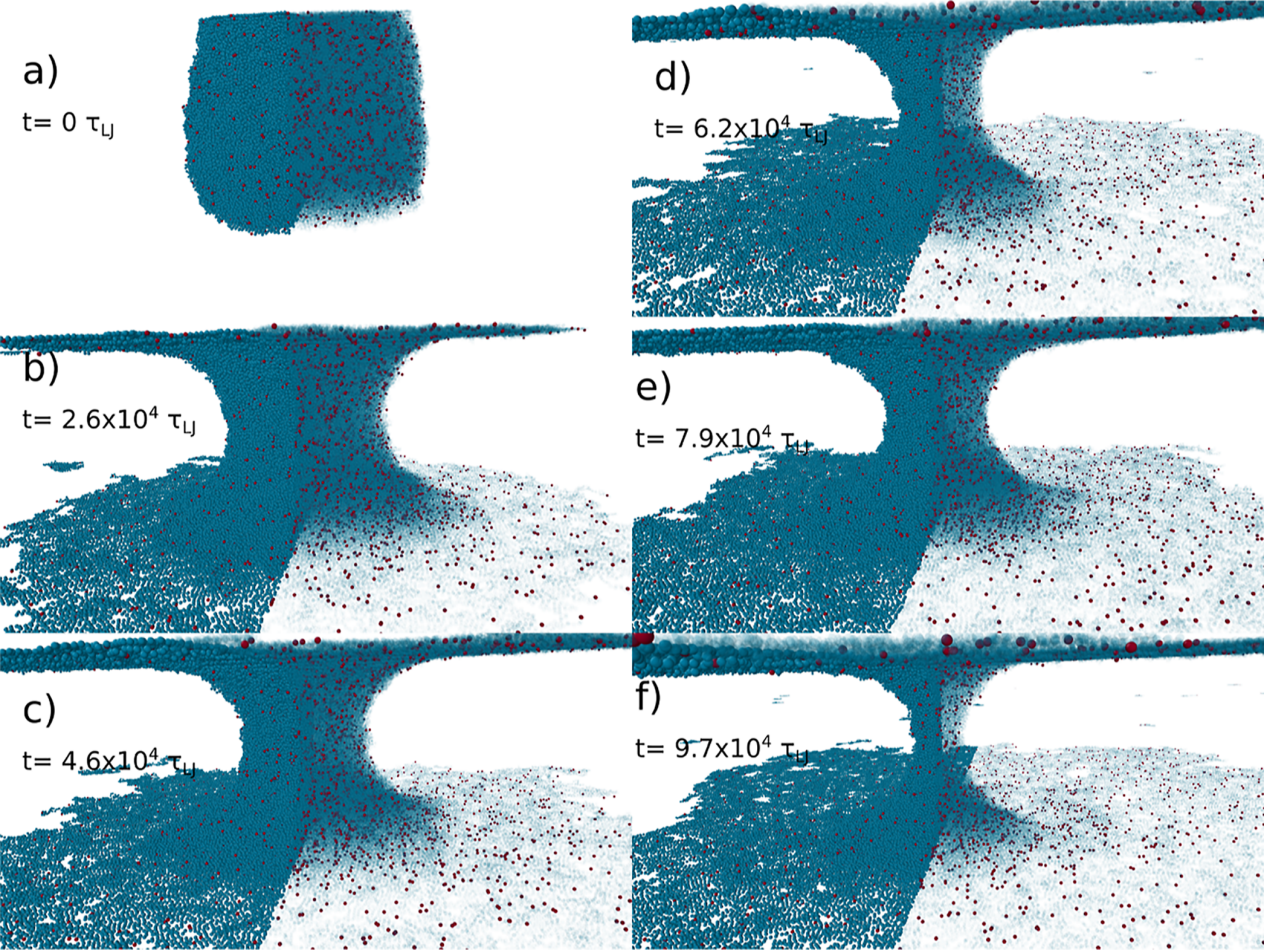
Temporal evolution of the stalk between the two plates in the KG model description. From the initial stalk (a) through a linearly thinning stalk (b–e) and finally to the exponential decay (f). For each snapshot: the left shows the polymer particles, while on the right, these are made transparent to highlight the entanglements in red. In the KG model, entanglements correspond to the kinks in the Z1+ analysis.

**Figure 5:**
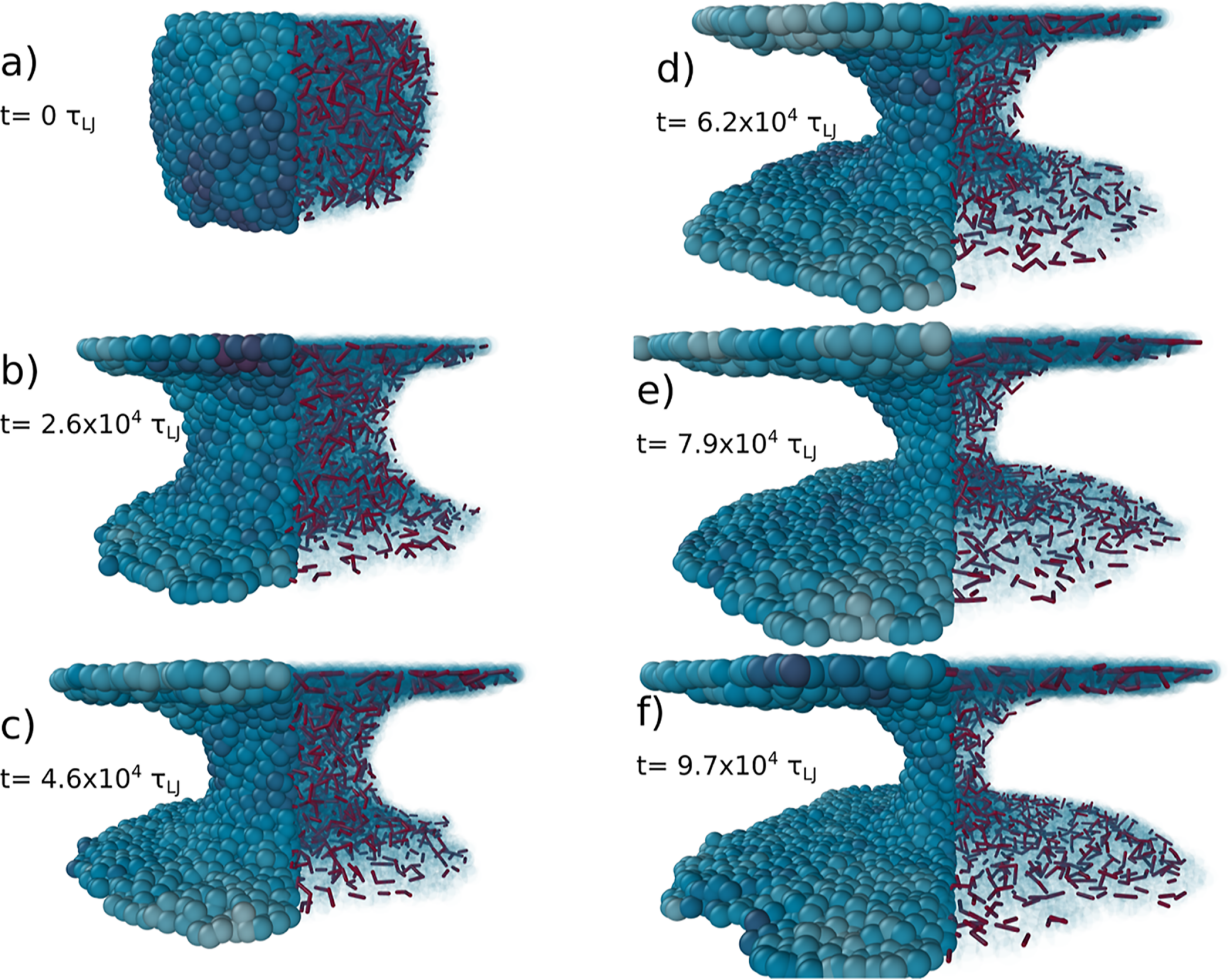
Time evolution of the stalk between the two plates in the SLSP model description. From the initial stalk (a) through a linearly thinning stalk (b–e) and finally to the exponential decay (f). For each snapshot: the left image shows the polymer particles. In the right image, the particles are transparent, to highlight the entanglements in red. In the SLSP model, entanglements are visualized as SLSP bonds.

In our simulations, we focus on a dense entangled polymer melt, which differs slightly from experimental setups that involve longer length scales. To investigate the behavior of the proposed models, we prepare a polymer melt droplet between two smooth walls with a LJ potential, separated by a distance of approximately 15.2*R*_e,0_. We use two different models: the KG model with wall parameters ε_wall,KG_ = 3.75 and a cutoff distance of 1σ_KG_ and the MDPD model with wall parameters ε_wall,DPD_ = 7.75 and a cutoff distance of 1σ_DPD_. These choices of parameters yield approximately the same initial diameter of the cylinder, *R*(0) ≈ 4.7*R*_e,0_. Figures 4a and 5a show the initial configuration. Since we have the same number of molecules and hence the same volume, by having the same droplet radius, we can implicitly match the ratio of surface tension between the vapor, liquid, and substrate. Note that MDPD simulations have been employed before to study wetting dynamics.^67^ Note that, in contrast to the established CaBER method, we do not extend the distance between the plates; instead, we control the wetting strength to induce capillary breakup (the CaBR method).

**Figure 6:**
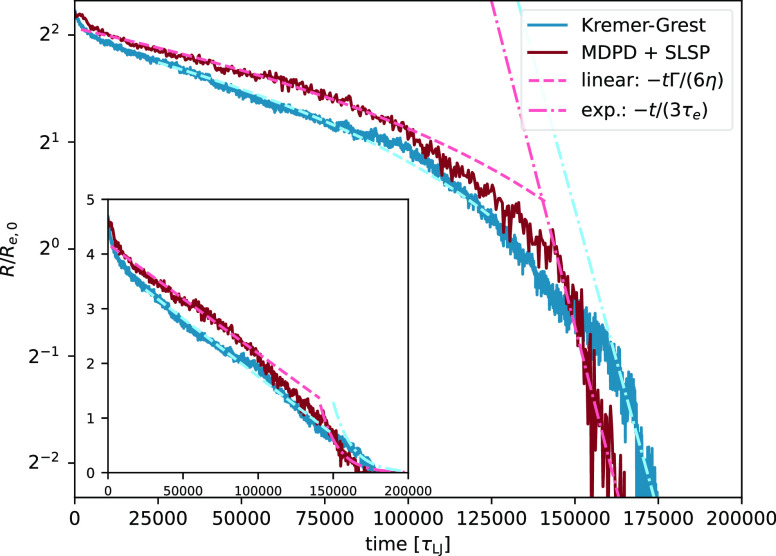
Neck size evolution of the CaBR meniscus during breakup as a function of time. The blue curve corresponds to the KG model, while the red curve corresponds to the SLSP model. Both models exhibit the characteristic breakup regimes predicted by theory and observed in experiments, and the inset emphasizes the initial linear decay.

At the beginning of a simulation, the attraction between the polymer and the two plates is increased instantaneously by setting ε_wall,KG_ = 6.75 and ε_wall,DPD_ = 15.0, which initiates the spreading of the material and the breakup of the cylinder element into two droplets that wet the two surfaces. In Figures 4b and 5b, one can appreciate the initial meniscus that is formed by the confined droplet in the two models. When wetting begins, in the initial stages, the neck size decays linearly with time, as predicted by theory. The KG filament radius decays slightly faster than that of the SLSP model, as shown in Figure 6. This difference is likely due to the fact that we matched the viscosity, η, implicitly between the models in the initial setup but did not explicitly match the surface tension, Γ. Therefore, a slight difference in the predicted linear regime *R*(*t*) ∝ −*t*Γ/(6η) is to be expected. Note that it should also be possible to modify the internal attraction for the MDPD model via *A*_*ij*_, *B* to ensure complete compatibility between the models. As the neck size diminishes to *R*(*t*) < 2*R*_e,0_, both models transition into the terminal regime. In this phase, the entanglement time τ_e_ becomes the pivotal factor influencing the dynamics of the filament. Both models display an exponential decay; the relaxation time for the SLSP model is denoted by τ_e,SLSP_ = (1.19 ± 0.9)10^4^τ_LJ_, and for the KG model, it is τ_e,KG_ = (1.27 ± 0.8)10^4^τ_LJ_. These values are derived from the lines fitted to the data in Figure 6.

In this entanglement-centric terminal regime, the two models are in remarkable quantitative agreement. This is evident not only in the timing sequence of the neck breakup process but also in the prefactors corresponding to theoretical predictions. This alignment reinforces the view that the MDPD model, with its explicit liquid–vapor phase, can seamlessly integrate with the SLSP model using bulk parameters, without necessitating any additional modifications.

Moreover, we observe that the SLSP model is in particularly good qualitative agreement with theoretical expectations, suggesting that the MDPD approach with SLSP can be used in CaBR-based simulations to understand rheological measurements and for direct interpretation of the experimental data. This also opens up the possibility of simulating nano-rheology CaBR experiments to determine rheological properties from digital twins. We recognize that creating complete digital twins will require advances in experimental measurement techniques. Specifically, the experimental determination of surface tensions becomes critical. One prospective method involves using height-to-width ratios of nano-droplets through the implementation of atomic force microscopy (AFM). It is important to note, however, that current access to capillary breakup measurements for nano-droplets is still limited. With new developments in these domains, it should be possible to arrive at faithful reconstructions of high-precision experiments.

## Conclusions

In conclusion, we have successfully extended the SLSP model originally proposed by Chappa *et al.*(4) to describe heterogeneous systems. Specifically, this expanded model can effectively describe the dynamics of entangled polymers in scenarios involving liquid–vapor or liquid–solid interfaces. To ensure coexistence while maintaining computational efficiency, we incorporated MDPD non-bonded interactions. With the introduction of a compensating potential to offset the attractive effects of additional springs in the bulk phase, the SLSP model can operate in tandem with the MDPD method. This integration facilitates the simulation of high-molecular-weight polymers, where chain conformations are regulated by a liquid–vapor interface and entanglements significantly influence outcomes.

The proposed method, extended through a CaBR-like approach, can encompass a broad spectrum of rheological properties in complex fluids, paving the way for direct comparisons to experimental systems.

The proposed MDPD + SLSP model’s validity has been confirmed through comparison to theoretical results and to the results of a fully entangled polymer model employing hard LJ interaction sites. A key feature of our proposed model is its ability to model evaporation, thereby enabling comprehensive studies of entangled dynamics during non-equilibrium processes involving high vapor-pressure solvents.

In conjunction with previously established high-detail models,^15^ our proposed approach provides a foundation for the development of digital twins of experimentally relevant liquid polymer melt systems. This includes micro-rheometers based on droplet deformation, such as CaBR.

## Supporting Information Available

Additional information comparing the KG and SLSP models (PDF)
