# Supplementary material for: Entanglements via Slip Springs with Soft, Coarse-Grained Models for Systems Having Explicit Liquid–Vapor Interfaces
Source: Macromolecules. 2023 Sep 1;56(18):7445–53. doi: 10.1021/acs.macromol.3c00960 (PMC10538480; doi:10.1021/acs.macromol.3c00960)
Supplement: Supplementary file 1 — ma3c00960_si_001.pdf [file ma3c00960_si_001.pdf]

Supporting Information: Entanglements via Slip-Springs with Soft, Coarse-Grained Models for Systems Having Explicit Liquid-Vapor Interfaces

Ludwig Schneider and Juan de Pablo*

Pritzker School of Molecular Engineering, University of Chicago, 5740 S. Ellis Ave, Chicago, IL, USA

E-mail: depablo@uchicago.edu

We use the end-to-end vector correlation function as published in Ref.[1 1]. We calculate the end-to-end vector correlation in both the Kremer-Grest and many-body dissipative particle dynamics (MDPD) + slip-spring (SLSP) model. Overlapping the correlation decay by adjusting a time matching factor τ between the models allows us to determine the time scale. Additionally, by using matching point at short and long time scales, a best match can be found over varying the SLSP fugacity z. Figure S1 shows this matching at short time scales, and it is straight forward to see, that $z = 0.225$ offers the best match at all time scales. The resulting time scale matching between the models is $\tau \approx 64$. In combination with the fact, that the SLSPs model is integrated with an order of magnitude larger time steps, the speed up between the models is three orders of magnitude without even considering the lower degrees of freedoms in the more coarse-grained model.

[1]Compare specifically with Figure 8a in Ref.[1]

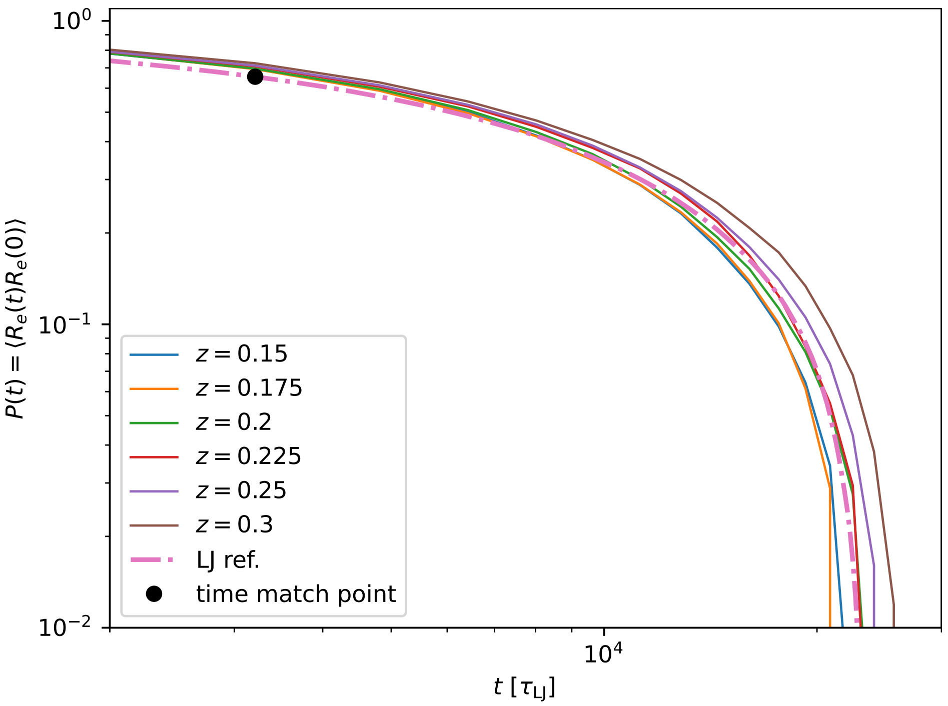

Figure S1: End-to-end vector correlation from the Kremer-Grest model and the equivalent MDPD + SLSP. We match the curves at different fugacities and overlap them at long and short time scales. Here the best match can be obtained for $z = 0.225$.

References

(1) Behbahani, A. F.; Schneider, L.; Rissanou, A.; Chazirakis, A.; Bacova, P.; Jana, P. K.; Li, W.; Doxastakis, M.; Polinska, P.; Burkhart, C., et al. Dynamics and rheology of polymer melts via hierarchical atomistic, coarse-grained, and slip-spring simulations. *Macromolecules* **2021**, *54*, 2740–2762.
